# Screening for Liver Fibrosis and Steatosis in a Large Cohort of Patients with Type 2 Diabetes Using Vibration Controlled Transient Elastography and Controlled Attenuation Parameter in a Single-Center Real-Life Experience

**DOI:** 10.3390/jcm9041032

**Published:** 2020-04-06

**Authors:** Ioan Sporea, Ruxandra Mare, Alina Popescu, Silviu Nistorescu, Victor Baldea, Roxana Sirli, Adina Braha, Alexandra Sima, Romulus Timar, Raluca Lupusoru

**Affiliations:** 1 Department of Gastroenterology and Hepatology, “Victor Babes” University of Medicine and Pharmacy, 300041 Timisoara, Romania; isporea@umft.ro (I.S.); alinamircea.popescu@gmail.com (A.P.); nissilviu@gmail.com (S.N.); victorbaldea07@gmail.com (V.B.); roxanasirli@gmail.com (R.S.); raluca_lupusoru@yahoo.ro (R.L.); 2 First Department of Internal Medicine, “Victor Babes” University of Medicine and Pharmacy, 300041 Timisoara, Romania; braha.adina@gmail.com; 3 Second Department of Internal Medicine, “Victor Babeș” University of Medicine and Pharmacy, 300041 Timișoara, Romania; alexa_moisuc@yahoo.com (A.S.); timarrz@yahoo.com (R.T.); 4 Department of Functional Sciences, “Victor Babes” University of Medicine and Pharmacy, 300041 Timisoara, Romania

**Keywords:** type 2 diabetes mellitus, liver fibrosis, steatosis, FibroScan measurements

## Abstract

Background: Type 2 diabetes mellitus (T2DM), obesity, hyperlipidemia, and hypertension are considered risk factors for developing non-alcoholic fatty liver disease (NAFLD). This study aims to assess steatosis and fibrosis severity in a cohort of T2DM patients, using vibration controlled transient elastography (VCTE) and controlled attenuation parameter (CAP). Material and method: We performed a prospective study in which, in each patient, we aimed for 10 valid CAP and liver stiffness measurements (LSM). To discriminate between fibrosis stages, we used the following VCTE cut-offs: F ≥ 2–8.2 kPa, F ≥ 3–9.7 kPa, and F4 - 13.6 kPa. To discriminate between steatosis stages, we used the following CAP cut-offs: S1 (mild) – 274 dB/m, S2 (moderate) – 290dB/m, S3 (severe) – 302dB/m. Results: During the study period, we screened 776 patients; 60.3% had severe steatosis, while 19.4% had advanced fibrosis. Female gender, BMI, waist circumference, elevated levels of AST, total cholesterol, triglycerides, blood glucose, and high LSM were associated with severe steatosis (all *p*-value < 0.05). BMI, waist circumference, elevated levels of AST, HbA1c, and CAP were associated with advanced fibrosis (all *p*-values < 0.05). Conclusion: Higher BMI (obesity) comprises a higher risk of developing severe steatosis and fibrosis. Individualized screening strategies should be established for NAFLD according to different BMI.

## 1. Introduction

The incidence of overweight and obesity has been increasing in the past decades in developed countries. Currently, more than one billion persons have a body mass index (BMI) higher than 25 kg/m^2^ [1]. High-calorie intake, increasing use of carbohydrates, and sedentary lifestyle are essential drivers of the global epidemic [2]. The epidemic of obesity affects mainly Northern America, Europe, and some areas of Asia. At the same time, the frequency of type 2 diabetes mellitus (T2DM) is increasing. About 1 of 11 adults worldwide have diabetes, 90% of them having type 2. Many patients with T2DM also have metabolic syndrome [3]. Furthermore, dyslipidemia is a common condition in obese and T2DM patients.

For a long time, diabetologists focused only on the “classic” complications of DM. However, in the last years, many papers underlined the severity of liver damage in these patients [4]. Obesity, together with T2DM (and sometimes with hypertriglyceridemia), leads to fat deposits in the liver, generating the non-alcoholic fatty liver disease (NAFLD), which can progress because of inflammation to non-alcoholic steatohepatitis (NASH) with progressive fibrosis. Such patients have an increased risk for developing cirrhosis, and sometimes, hepatocellular carcinoma. A recent study showed that the prevalence of NAFLD in type 2 diabetes is approximately 60% [5]. Other studies from France and Turkey showed that the presence of fibrosis is high in these subjects [6,7].

Many studies used liver biopsy to evaluate these patients, especially for the diagnosis of NASH. On the other hand, non-invasive methods are increasingly used in clinical practice for the evaluation of liver steatosis and fibrosis. Starting from simple biological scores such as APRI or FIB-4 (used mainly by general practitioners), to patented, complex ones (ELF, FibroMax, NAFLD score) and continuing with elastographic methods (ultrasound-based or MRI based), all of them trying to identify the pathological cases in a vast cohort of individuals with predisposing conditions.

Vibration controlled transient elastography (VCTE) (FibroScan^®^, EchoSens, Paris, France), developed more than 15 years ago, was used extensively for the evaluation of liver fibrosis in chronic liver diseases. With a rate of failed and unreliable measurements that can reach up to 30% in obese patients using only the standard M probe [8], the use of the XL probe in the obese can increase feasibility to more than 90% [9]. The addition of controlled attenuation parameter (CAP), used for the evaluation and quantification of liver fatty infiltration, makes FibroScan a valuable system for liver assessment in subjects at risk for NASH [10].

Screening the population at risk for liver steatosis and fibrosis is an important objective in daily practice, knowing that fibrosis is the main driver of prognosis in NAFLD patients [11]. Steatosis can be managed with lifestyle changes, as opposed to fibrosis, which is more challenging to address.

Our study aims to evaluate the incidence of steatosis and fibrosis and the factors associated with these conditions in a large cohort of T2DM patients, using vibration controlled transient elastography (VCTE) and controlled attenuation parameter (CAP).

## 2. Materials and Methods

### 2.1. Study Population

A prospective study was conducted between January 2017 and August 2018 in the Department of Gastroenterology and Hepatology and the Department of Diabetes and Metabolic Diseases in Timișoara Emergency County Hospital. We enrolled consecutive Caucasian patients, mostly elderly patients, scheduled for a medical visit in the Department of Diabetes and Metabolic diseases that agreed to be evaluated by elastography. All these patients underwent vibration controlled transient elastography (VCTE) and controlled attenuation parameter (CAP) screening.

Inclusion criteria were: patients older than 18 years, diagnosed with T2DM according to the American Diabetes Association criteria [12], willing to undergo VCTE and CAP measurements.

Exclusion criteria were: known chronic liver diseases (the following parameters were documented Hbs Ag, HCV Ab, gamma-glutamyl transpeptidase (GGT), alkaline phosphatase (AP), alcohol intake more than 20 g/day in women and >30 g/day in men, use of drugs that induce steatosis (e.g., tamoxifen, steroids), pregnancy, cardiac pacemakers, malignancy, end-stages renal diseases, heart failure, unreliable or invalid VCTE and CAP measurements, elevation of aspartate aminotransferase (AST) and alanine aminotransferase (ALT) more than five times the upper limit of normal (ULN) values and outliers (subjects that had inexplicable higher values at laboratory data). Primary or secondary hypertriglyceridemia or hypercholesterolemia were not excluded as these individuals have frequently associated these comorbidities.

All patients gave their informed consent for the procedure. The study protocol was conducted according to the Helsinki Declaration after the approval by our institution’s Ethical Committee number 375/24.03.2018.

### 2.2. Clinical Assessment

Clinical assessment, anthropometric, and demographic data were collected on the same day with the measurements by VCTE and CAP. Laboratory values (ALT, AST, GGT, thrombocytes, blood glucose, HbA1c, cholesterol, triglycerides) were measured within one month and collected from medical records. BMI was calculated as weight in kilograms divided by square of height in meters. Normal weight, overweight, and obesity were defined as BMI < 25 kg/m^2^, BMI between 25 and 30 kg/m^2^, and BMI ≥ 30 kg/m^2^, respectively. Alcohol intake was evaluated using the questionnaire for the AUDIT-C score.

### 2.3. Vibration Controlled Transient Elastography (VTCE) and Controlled Attenuation Parameter (CAP) Measurements

VCTE was performed with a FibroScan^®^ device (EchoSens, Paris, France) (Figure S1), in fasting conditions for more than 4 h, with the patient in a supine position, right arm in maximum abduction, by intercostal approach, in the right liver lobe. In each patient, we aimed for 10 valid liver stiffness measurements (LSM). The examination was performed using the M probe (standard probe – transducer frequency 3.5 MHz) or the XL probe (transducer frequency 2.5 MHz). M and XL probes were used according to the European recommendation on M and XL probe selection [13]. A median value of 10 valid LSM was calculated, and the results were expressed in kilopascals (kPa). Reliable measurements were defined as the median value of 10 valid LSM with an interquartile range interval/median ratio (IQR/M) < 30% [8,14,15]. To discriminate between the stages of fibrosis, we used the following TE cut-offs: F ≥ 2–8.2 kPa, F ≥ 3–9.7 kPa, and F4 - 13.6 kPa [16].

To discriminate between steatosis stages, we used the following CAP cut-offs: S1 (mild) – 274 dB/m, S2 (moderate) – 290 dB/m, S3 (severe) – 302 dB/m [16].

### 2.4. Surogate Serum Fibrosis Markers

For each patient APRI score [17] and FIB-4 score [18] were determined by using the following criteria:

APRI = ((aspartate aminotransferase (U/L) / aspartate aminotransferase upper limit of normal (U/L)) × 100)/platelet count [10⁹/L).

FIB-4 = (age (years) × aspartate aminotransferase (U/L)/(platelet count (10⁹/L) × square root of alanine aminotransferase (U/L)).

### 2.5. Statistical Analysis

All statistical tests were performed using R software V.2.5.1 (R Development Core Team, Vienna, Austria) and IBM SPSS Statistics V.17 (IBM Statistics, Chicago, IL, USA). The Kolmogorov–Smirnov test was used for testing the distribution of numerical variables. Qualitative variables were presented as numbers and percentages. Parametric tests (*t*-test, ANOVA) were used for the assessment of differences between numerical variables with normal distribution; and nonparametric tests (Mann-Whitney or Kruskal-Wallis tests) for variables with non-normal distribution. The Chi-square (χ^2^) test was used for comparing proportions expressed as percentages (“*n*” designates the total number of patients included in a particular subgroup). Linear and logistic regression were used for univariate and multivariate analysis of factors that may influence LSM and CAP values. Pearson correlation coefficient (r) was used in order to evaluate the association between two variables. Furthermore, 95% confidence intervals were calculated for each predictive test, and a *p*-value < 0.05 was considered significant for all statistical tests.

## 3. Results

### 3.1. Baseline Characteristics

**Figure 1:**
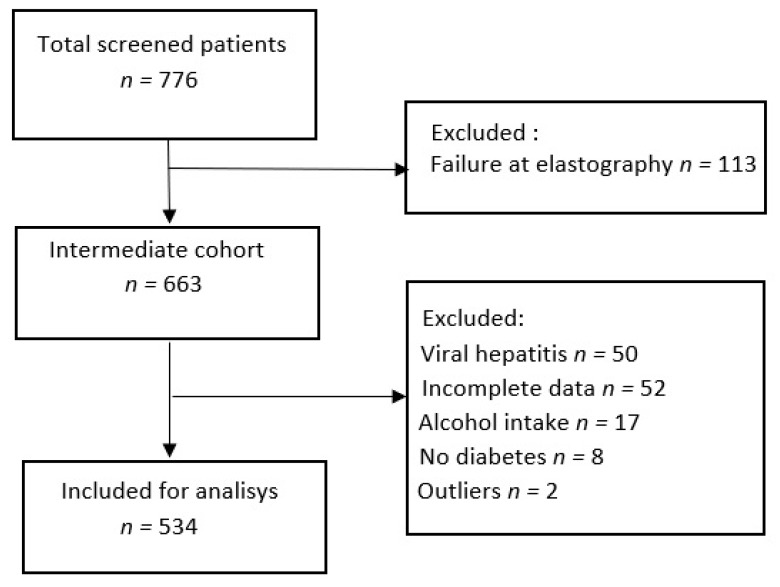
Study flow diagram. A total of 242 patients were excluded from the study.

A total of 776 patients were screened using VCTE and CAP during the study period, 242 of them were excluded because of (Figure 1) invalid VCTE measurements—14.5% (113) patients (because of obesity, *p* < 0.0001), associated chronic viral hepatitis–6.4% (50) patients, incomplete clinical and laboratory data–6.7% (52) patients, high alcohol consumption–2.1% (17) subjects, absence of T2DM–1% (8) patients, and 2 (0.4%) outliers. We analyzed the two groups, the study group and the patients that were excluded, in order to see the differences between them (Table 1). BMI, waist circumference, transaminases, LSM and CAP values were higher in the excluded group than in the study group. Insulin treatment and liver cirrhosis were found more frequently in the study group.

Finally, a total of 534 subjects were included in the analysis. Group characteristics are presented in Table 2.

In the study cohort of 534 T2DM patients, the distribution of steatosis severity assessed by CAP was as follows: 23.9% (127) patients had no steatosis–S0, 8.9% (48) had S1, 6.9% (37) S2, and 60.24% (322) patients S3. There was no significant difference among genders (Table 2).

Regarding fibrosis severity, according to VCTE measurements, 72.6% (388 patients) had no or mild fibrosis–F0 and F1, 7.8% (42) had F2, 11.4% (61) F3, and 8.2% (43 patients) F4. There was no significant difference among fibrosis severity by VCTE according to gender distribution (Table 2).

Furthermore, we divided our final cohort into three groups–normal weight, overweight, and obese—and we found that the mean LSM was significantly higher in obese patients (6.92 ± 5.85 kPa versus 7.21 ± 2.1 kPa versus 8.31 ± 6.4 kPa, respectively, *p* = 0.03). Also, the mean CAP values were significantly higher in patients with excessive weight (255.56 ± 60.8 dB/m versus 300.9 ± 55.8 dB/m versus 335.2 ± 51.2 dB/m, respectively, *p* < 0.0001).

Patients with at least advanced fibrosis was found more frequently in obese patients than in overweight and normal-weight patients, 23.3% vs. 14.1% vs. 12%, respectively, *p* < 0.001.

The absence of steatosis was strongly correlated with normal weight (*r* = 0.90, *p* = 0.01); mild steatosis was correlated with overweight (*r* = 0.69, *p* < 0.0001), and severe steatosis were strongly correlated with obesity (*r* = 0.91, *p* < 0.0001).

### 3.2. Factors Associated with Severe Steatosis at CAP

CAP values significantly increased with weight status. For the entire cohort we found that female gender (*p* = 0.02), BMI (*p* = 0.03), waist circumference (*p* < 0.0001), elevated levels of AST (*p* = 0.03), total cholesterol (*p* = 0.01), triglycerides (*p* < 0.0001), blood glucose (*p* = 0.0009), and high LSM (*p* = 0.0006) were associated with severe steatosis (Table 3). However, in multivariate analysis, none of them were independently associated with severe steatosis (Table 4).

In the normal-weight group, also female gender (*p* = 0.01), increased waist circumference (*p* = 0.006), triglycerides (*p* < 0.0001), and LSM (*p* = 0.02) were associated with severe steatosis, but none was independently associated (Table 4).

In the overweight group, only increased waist circumference (*p* = 0.02) and elevated level of total cholesterol (*p* = 0.03) were associated with severe steatosis, but also not independently.

In the obese group, elevated BMI (*p* = 0.0008), waist circumference (*p* = 0.001), AST (*p* = 0.001), triglycerides (*p* = 0.07), blood glucose (*p* = 0.001), and HbA1c (*p* = 0.008) were associated with severe steatosis. In multivariate analysis, only waist circumference was independently associated with severe steatosis (*p* = 0.002).

### 3.3. Factors Associated with Advanced Fibrosis (F3) by VCTE

LSM increased with increasing BMI and waist circumference. For the entire cohort, we found that BMI (*p* < 0.0001), waist circumference (*p* = 0.0002), an elevated level of AST (*p* < 0.0001), severe steatosis (0.0007) HbA1c (*p* = 0.04), and higher CAP values (*p* = 0.002) were associated with advanced fibrosis (F3 and F4) (Table 5). In multivariate analysis, only AST was independently associated with advanced fibrosis (Table 6).

In the normal-weight group, the female gender (*p* = 0.006), AST (*p* = 0.01), and CAP (*p* = 0.01) were associated with advanced fibrosis, but only AST was independently associated (Table 6).

In the overweight group, only an elevated level of AST (*p* = 0.002) was associated with advanced fibrosis.

In the obese group, BMI (*p* < 0.0001), waist circumference (*p* = 0.002), an elevated level of AST (*p* = 0.01), severe steatosis (*p* = 0.03), and the elevated level of HbA1c (*p* = 0.02) were associated with advanced fibrosis. In multivariate analysis, only AST (*p* = 0.001) and severe steatosis (*p* < 0.0001) were independently associated with advanced fibrosis. The risk for advanced fibrosis was 6.5 times higher in patients with severe steatosis (OR = 5, 95% CI: 1.5–31.4, *p* < 0.0001).

### 3.4. Factors Associated with Significant Fibrosis (F2) by VCTE

For significant fibrosis, we found that only BMI and waist circumference were associated with significant liver fibrosis in overall patients and in obese patients (*p* < 0.001 and *p* = 0.006 for BMI and *p* = 0.0002 and *p* = 0.03 for waist circumference), but not independently (all *p*-values > 0.05).

### 3.5. Comparison of Transient Elastography with FIB-4 and APRI

There was a significant difference between LSM obtained with transient elastography, APRI, and FIB-4 among patients with mild and significant fibrosis (≤F2) and those with advanced fibrosis (F ≥ 3) (Table 7).

**Figure 2:**
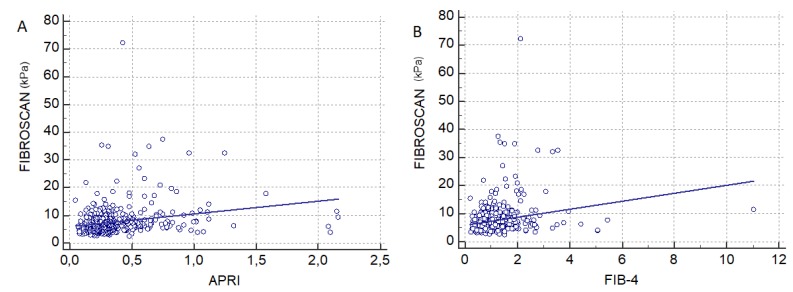
(**A**) Correlation between LSM obtain with Fibroscan and APRI score (*r* = 0.22, *p* < 0.0001). (**B**) Correlation between LSM obtained with Fibroscan and FIB-4 score (*r* = 0.21, *p* < 0.0001).

For overall patients, we found a direct, weak, but extremely significant association between transient elastography and APRI (*r* = 0.22, *p* < 0.0001) (Figure 2A) and between transient elastography and FIB-4 (*r* = 0.21, *p* < 0.0001) (Figure 2B. For advanced fibrosis the correlation coefficient between TE and APRI was *r* = 0.15, *p* = 0.01 and between TE and FIB4, *r* = 0.20 (*p* < 0.0001). For mild and significant fibrosis (F ≤ 2), the correlation coefficient between TE and APRI was *r* = 0.15, *p* < 0.001 and between TE and FIB4, *r* = 0.15, *p* < 0.0001.

## 4. Discussion

The problem of NAFLD in the general population and also in particular categories, such as patients with T2DM or metabolic syndrome, has become a subject of extensive research in the last decades. Several papers and meta-analysis underlined the importance of NAFLD in T2DM patients [1,6,19], but the medical community is not prepared yet to start screening for NAFLD and NASH in all diabetic patients. This paper would like to point out, for all the healthcare givers involved in this field (diabetologists, internal medicine specialists, and hepatologists), that this pathological condition is quite frequent in the daily practice. It seems that there is an association between a high amount of body fat in T2DM and the incidence of NAFLD. Therefore, we should insist on lifestyle changes through diet and physical activity in this category of patients.

Published data show that the prevalence of NAFLD varies between 42.6 and 69% in T2DM patients [20,21], while a previous study from our area showed a prevalence of NAFLD in T2DM patients of up to 87.1% [22]. The prognosis of these patients is different if they present only simple steatosis—non-alcoholic fatty liver (NAFL)—or if they have already developed NASH, early NASH (no or mild fibrosis), fibrotic NASH (significant/advanced fibrosis), or NASH cirrhosis [23].

How to screen for fatty liver? The easiest, cheapest, and most commonly available way is liver ultrasound examination (US). The accuracy of this method for the detection of moderate and severe steatosis is quite high—more than 80% in a meta-analysis compared to that of liver biopsy [20]. However, the quantification of steatosis by the US is a subjective method, and the medical community would like to have more objective, numeric values. For this reason, CAP by FibroScan evolved to be a reference method in the last years. Several studies demonstrated that the accuracy of CAP in comparison with liver biopsy is higher than 80% [21,22], and this is quite enough for daily practice. The introduction of CAP on both M and XL probes of FibroScan increased the feasibility of the method [23]. In the last years, some problems were raised regarding CAP, mainly concerning the proposed cut-off values in different categories of patients [24,25] and regarding the need to use quality criteria for the technique [26].

Considering the large number of patients at risk, which need to be evaluated, and the search for accurate non-invasive assessment methods, proton density fat fraction (PDFF) by MRI recently became an alternative to liver biopsy for the repetitive quantification of liver steatosis [26,27]. Furthermore, encouraging results of quantification techniques for steatosis implemented on ultrasound systems have been published [28,29,30,31]. Currently, VCTE is one of the reference methods used for liver fibrosis assessment in all liver diseases [14,32], but other ultrasound-based elastography techniques (point SWE or 2D-SWE) and magnetic resonance-elastography (MR-E) are also used in clinical practice. The proposed cut-off values for staging fibrosis by VCTE vary according to the etiology (viral hepatitis, NAFLD, others), and also according to the type of probe used (M vs. XL). Although we used the M and XL probes, the feasibility of VCTE was only 85.5%, less than that reported in a previous study [9], possibly because of the high number of obese diabetic patients (more than 60%, with a mean BMI of 35.5 kg/m^2^).

We consider that the most relevant result of our study is the underlining of the high prevalence of liver steatosis (mainly severe) in T2DM patients. Probably mild steatosis has no significant clinical relevance, but moderate and severe steatosis can lead to severe liver damage over time. In our study, in univariate analysis, female gender, BMI, waist circumference, elevated level of AST, total cholesterol, and triglycerides, high blood glucose values were associated with severe steatosis. Still, in multivariate analysis, none of them was independently associated with severe steatosis. From the univariate analysis, we can speculate which of the T2DM patients are at the highest risk for developing NAFLD and which ones should be selected for screening. In our cohort the levels of triglycerides were high ranging from 30 to 8000mg/dl. Hypertriglyceridemia is frequently associated to T2DM as well as hypercholesterolemia. One explanation for this wide range of triglycerides is that at the time of elastographic assessment, some patients were already known with these comorbidities (and undergoing treatment) and some of them were not, and thus untreated.

Liver fibrosis severity is the main prognostic factor in NAFLD patients [11,33]. When we evaluated fibrosis severity by VCTE in our group of patients, based on the cut-offs proposed by Eddowes et al. [16], we found that 19.4% of these patients had a high risk for developing severe fibrosis (11.3% had F3 and 8.1% F4), thus having chronic advanced compensated liver disease (cACLD) and being at risk for portal hypertension, decompensated liver disease or development of hepatocellular carcinoma. Because almost 20% of the diabetic patients are at risk for cACLD, it seems reasonable to screen all diabetic patients by liver elastography.

In our cohort, in univariate analysis, BMI, waist circumference, an elevated level of AST, severe steatosis, and high level of HbA1c were associated with advanced fibrosis. In multivariate analysis, only AST and severe steatosis were independently associated with advanced fibrosis. We also found that patients with severe steatosis were at 6.5 times higher risk for advanced fibrosis (OR = 5, 95% CI: 1.5–31.4, *p* < 0.0001). Using these results of the univariate and multivariate analysis, we can conclude that, in our cohort, the group with the highest risk for advanced fibrosis consists of obese diabetic patients, with high waist circumference, elevated AST, severe steatosis, and uncontrolled diabetes (elevated HbA1c).

Our study has some limitations. The first one is that the evaluation of steatosis and fibrosis in our cohort was performed only non-invasively, without performing a liver biopsy, which is the gold standard in this situation. Second, we did not continue the assessment of patients with increased AST and advanced fibrosis by liver biopsy to discriminate between NAFLD and NASH. Also, we did not use different cut-off values for fibrosis assessment according to the probe used (M vs. XL). However, a recent study showed that if the right probe is used, selected by the automatic tool of the device, based on the skin to liver distance, there are no significant differences between liver stiffness values obtained by the M and XL probes [34]. Regarding transient elastography limitation, 14.5% of the patients were excluded from the final analysis because of their unreliable results. Obesity was the only factor associated with TE failure. On the other hand, the patients excluded from the study had higher transaminases, higher liver stiffness, and higher CAP values due to the additional risk factors for chronic liver disease (hepatitis viruses, chronic infection and/or alcohol abuse). Two patients were excluded from the study, because they were considered outliers—they had very high triglycerides values without any explanation. The two had severe steatosis at CAP and were F0-F1 at TE.

APRI score and FIB-4 score can rule out advanced fibrosis, but these two simple score, together with TE could be used as first line tests to rule out diabetes patients with advanced fibrosis. Similar to other studies [35,36,37], the performances of APRI score and FIB-4 score were comparable to those obtained with Fibroscan. Higher APRI and FIB-4 scores were associated with higher VCTE values, while lower APRI and FIB-4 scores were associated with lower VCTE values.

Finally, which are the main messages of our paper? Many T2DM patients are at high risk of developing severe steatosis and advanced fibrosis, and we believe that it is highly recommendable for them to be screened for chronic liver disease. This screening should be performed in all patients, or, at least, in those presenting more risk factors. VCTE and CAP are good methods for NASH screening, but, probably, new ultrasound systems able to quantify steatosis and fibrosis severity by different elastography techniques, would be even more accurate. This paper is in line with a Turkish study [7] that included 124 T2DM patients. The prevalence rates of obesity were similar (64.5% vs. 61.2%, *p* = 0.5), and in both studies, approximately 20% of patients had at least advanced fibrosis. Similar results were obtained in a French study [6] that included 705 T2DM patients, in whom higher BMI was associated with higher LSM and CAP measurements. Also, in a larger Dutch cohort [19], higher LSM were strongly associated with steatosis and T2DM. These three studies [6,7,19] showed that diabetic patients are at high risk for steatosis and significant fibrosis. The differences in the prevalence among the three groups can be explained by the different prevalence of obesity among the groups and by the quality of diabetes control.

## 5. Conclusions

Total of sixty percent of the T2DM patients in our cohort had severe steatosis, evaluated by CAP, and almost 20% of them had advanced fibrosis, measured by VCTE. A higher BMI comprises a greater risk of developing severe steatosis and fibrosis. Individualized screening strategies should be established for NAFLD, according to BMI.

## Figures and Tables

**Table 1:** Comparison between excluded patients and study group.

Parameter	Study Group (*n* = 534)	Excluded Group (*n* = 242)	*p*-Value
Age, years (means)	60.8 ± 8.7	61.5 ± 7	0.27
Gender (*n*,%)			
Male	251 (47.1%)	87 (35.9%)	0.004
Female	283 (52.9%)	155 (64.1%)	0.004
BMI (kg/m^2^) (mean ± SD)	32 ± 6	35.01 ± 6	<0.0001
Hypertension (*n*,%)	295 (55.2%)	131 (54.1%)	0.83
Waist circumference (median, range)	108 (68–148)	110 (92–155)	<0.01
AST, IU/L (median, range)	23 (7–150)	36 (20–152)	<0.0001
ALT, IU/L (median, range)	36 (9–160)	50.4 (14.8–176)	<0.0001
Platelets × 10^3^/mm^3^ (median, range)	242 (71–450)	234 (67–602)	0.83
Total cholesterol, mg/dL (median, range)	179 (70–418)	182 (87–879)	0.65
Triglycerides, mg/dL (median, range)	149 (30–887)	220 (29–8000)	<0.001
LDL, mg/dL (median, range)	105 (7–262)	107(34–277)	0.37
HDL, mg/dL (median, range)	35 (10–120)	40 (13–120)	0.58
LSM, kPa (mean ± SD)	7.73 ± 5.7	13.2 ± 7.1	<0.0001
CAP, dB/m (mean ± SD)	317 ± 59.5	336 ± 61.1	<0.0001
Fibrosis stage	*n* = 534	*n* = 69	
F0-1	388 (72.6%)	26 (37.6%)	<0.0001
F2	42 (7.8%)	6 (10.1%)	0.35
F3	61 (11.4%)	6 (10.1%)	0.68
F4	43 (8.2%)	31 (42.2%)	<0.0001
Steatosis stage	*n* = 534	*n* = 69	
S0	127 (23.9%)	19 (27.5%)	0.32
S1	48 (8.9%)	5 (7.2%)	0.51
S2	37 (6.9%)	4 (5.7%)	0.63
S3	322 (60.3%)	41 (59.6%)	0.91
Insulin	106 (19.7%)	79 (32.9%)	0.0001
Oral antidiabetics	328 (61.4%)	107 (44.2%)	<0.0001
T2DM duration	10 ± 2.0	15 ± 4.1	<0.0001

**Table 2:** Baseline characteristics of the study group according to weight condition.

Parameter	Normal Weight (*n* = 57)	Overweight (*n* = 150)	Obesity (*n* = 327)	*p*-Value
Age, years (means)	62 ± 8.6	61.1 ± 10.3	59.7 ± 9.71	0.09
Gender (*n*,%)				
Male	25 (43.8%)	72 (48%)	154 (47.1%)	0.75
Female	32 (56.2%)	78 (52%)	173 (52.9%)	0.75
BMI (kg/m^2^) (mean ± SD)	22.8±1.9	27.7±1.4	35.5±4.6	<0.0001
Hypertension (*n*,%)	30 (52.63%)	77 (50.9%)	188 (57.3%)	0.63
Waist circumference (median, range)	90 (68–110)	100 (70–118)	115 (90–148)	0.75
AST, IU/L (median, range)	23 (12–132)	21 (9–136)	24 (7–150)	0.42
ALT, IU/L (median, range)	34 (14–120)	36 (13–143)	37 (9–160)	0.98
Platelets × 10^3^/mm^3^ (median, range)	242 (78–418)	236 (71–441)	245 (82–602)	0.50
Total cholesterol, mg/dL (median, range)	184 (96–288)	186 (70–400)	194 (77–418)	0.08
Triglycerides, mg/dL (median, range)	141 (30–582)	146 (50–598)	160 (43–887)	0.10
LDL, mg/dL (median, range)	114 (7–205)	107 (12–215)	110 (17–262)	0.35
HDL, mg/dL (median, range)	47 (25–120)	41 (7–121)	40 (10–131)	0.51
LSM, kPa (mean ± SD)	6.92 ± 5.85	7.21 ± 2.1	8.32 ± 6.34	0.03
CAP, dB/m (mean ± SD)	255.56 ± 60.8	300.9 ± 55.8	335.2 ± 51.2	<0.0001
Fibrosis stage				
F0-1	45 (78.9%)	121 (80.6%)	222 (67.9%)	0.93
F2	4 (7%)	11 (7.3%)	29 (8.8%)	0.82
F3	5 (8.7%)	12 (8%)	42 (13%)	0.90
F4	3 (5.4%)	6 (4%)	34 (10.3%)	0.95
Steatosis stage				
S0	36 (63.1%)	45 (29.9%)	46 (14.1%)	<0.0001
S1	6 (10.5%)	19 (12.5%)	23 (7%)	<0.0001
S2	1 (1.9%)	12 (8%)	24 (7.4%)	0.2
S3	14 (24.5%)	75 (49.6%)	234 (71.5%)	<0.0001
Insulin	10 (18%)	46 (30%)	50 (52%)	<0.0001
Oral antidiabetics	22 (6.6%)	126 (38.1%)	182 (55.1%)	<0.0001
T2DM duration	8 ± 1.2	9 ± 2.3	13 ± 1.4	0.34

**Table 3:** Univariate analysis of factors associated with severe steatosis.

Variable	Overall			Normal Weight			Overweight			Obesity		
	ß	SE	*p*-Value	ß	SE	*p*-Value	ß	SE	*p*-Value	ß	SE	*p*-Value
Age	0.84	0.002	0.06	0.28	0.4	0.93	0.61	0.24	0.61	0.89	0.15	0.25
Female gender	–0.60	0.02	<0.0001	0.45	0.05	0.01	0.10	0.02	0.47	0.54	0.01	0.54
BMI	0.02	0.003	<0.0001	0.02	0.02	0.35	0.03	0.02	0.30	0.08	0.18	0.0008
Waist circumference	–0.69	0.17	<0.0001	0.01	0.006	0.006	0.36	0.41	0.02	0.14	0.2	0.001
AST	0.52	0.03	0.03	0.18	0.1	0.39	0.48	0.07	0.79	0.61	0.04	0.001
ALT	0.60	0.02	0.41	0.18	0.09	0.40	0.36	0.08	0.07	0.71	0.02	0.52
Total cholesterol	0.41	0.08	0.01	0.01	0.001	0.24	0.16	0.01	**0.03**	0.54	0.09	0.07
Triglycerides	0.43	0.04	<0.0001	0.002	0.002	<0.0001	0.37	0.06	0.09	0.63	0.04	0.007
Blood glucose	0.41	0.06	0.0009	0.25	0.16	0.91	0.32	0.1	0.09	0.47	0.07	0.001
HbA1c	0.37	0.1	0.06	0.03	0.01	0.83	0.35	0.2	0.55	0.36	0.1	0.008
LSM	0.50	0.03	0.0006	0.02	0.08	**0.02**	0.35	0.08	0.05	0.67	0.04	0.12
Insulin	0.60	0.03	0.10	0.31	0.06	0.06	0.53	0.05	0.20	0.67	0.04	0.12
Oral Antidiabetics	0.52	0.04	0.14	0.11	0.01	0.19	0.41	0.09	0.26	0.67	0.05	0.73
T2DM duration	0.67	0.03	0.10	0.22	0.09	0.94	0.56	0.08	0.65	0.1	0.02	0.54

ß = beta coefficient from regression analysis, SE = standard error.

**Table 4:** Multivariate analysis of factors associated with severe steatosis.

Variable	Overall		Normal Weight		Overweight		Obesity	
	OR 95% CI	*p*-Value	OR 95% CI	*p*-Value	OR 95% CI	*p*-Value	OR 95% CI	*p*-Value
Female gender	0.89(0.75–0.95)	0.85	0.59 (0.45–0.78)	0.78	–	–	–	–
BMI	0.99 (0.92–1.07)	0.97	0.89 (0.46–1.79)	0.76	–	–	1.02 (0.94–1.11)	0.14
Waist circumference	1.07 (1.03–1.11)	0.05	1.13 (0.97–1.32)	0.10	1.07 (1.005–1.14)	0.08	1.05 (0.98–1.08)	0.002
AST	1.01 (0.99–1.02)	0.11	–	–	–	–	0.99 (0.98–1)	0.10
Total cholesterol	1 (0.99–1.009)	0.70	–	–	1 (0.99–1.01)	0.17	–	–
Triglycerides	1 (1.002–1.009)	0.07	1.02 (1–1.14)	0.94	–	–	1.01 (0.97–1.04)	0.31
Blood glucose	1 (0.99–1.006)	0.22	–	–	–	–	1 (0.99–1)	0.32
HbA1c	–	–	–	–	–	–	1 (0.99–1.02)	0.27
LSM	1.08 (1.03–1.13)	0.58	1 (0.98–1.25)	0.68	–	–	–	–

CI = confidence interval, OR = odds ratio.

**Table 5:** Univariate analysis of factors associated with advanced fibrosis.

Variable	Overall			Normal Weight			Overweight			Obese		
	ß	SE	*p*	ß	SE	*p*	ß	SE	*p*	ß	SE	*p*
Age	0.16	0.1	0.75	0.20	0.3	0.85	0.004	0.002	0.10	0.26	0.14	0.83
Female gender	0.20	0.01	0.45	-0.24	0.08	0.006	0.18	0.01	0.32	0.05	0.04	0.21
BMI	0.01	0.002	<0.0001	0.03	0.02	0.09	0.02	0.01	0.2	0.25	0.02	<0.0001
Waist circumference	0.0006	0.001	0.0002	0.08	0.04	0.08	0.2	0.2	0.64	0.54	0.02	0.002
AST	0.004	0.009	<0.0001	0.05	0.02	0.01	0.05	0.01	0.0002	0.16	0.03	0.01
ALT	0.24	0.02	0.43	0.002	0.01	0.09	0.01	0.01	0.23	0.23	0.02	0.57
Total cholesterol	0.17	0.06	0.76	0.26	0.18	0.5	0.13	0.08	0.95	0.19	0.09	0.68
Triglycerides	0.0004	0.04	0.53	0.01	0.08	0.07	0.14	0.03	0.85	0.25	0.03	0.49
Blood glucose	0.31	0.06	0.13	0.24	0.12	0.30	0.14	0.07	0.91	0.19	0.07	0.49
HbA1c	0.02	0.01	0.04	0.39	0.2	0.21	0.01	0.01	0.3	0.03	0.01	**0.02**
CAP	0.001	0.002	0.0002	0.001	0.07	0.01	0.1	0.1	0.1	0.03	0.1	0.17
Severe steatosis	0.11	0.03	0.0007	0.52	0.34	0.13	0.35	0.5	0.47	0.58	0.1	0.03
Insulin	0.01	0.02	0.55	0.2	0.01	0.15	0.1	0.02	0.57	0.2	0.05	0.45
Oral Antidiabetics	0.15	0.02	0.28	0.2	0.02	0.8	0.05	0.01	0.47	0.84	0.01	0.89
T2DM duration	0.22	0.02	0.34	0.4	0.04	0.58	0.06	0.01	0.51	0.75	0.01	0.74

ß = beta coefficient from regression analysis, SE = standard error.

**Table 6:** Multivariate analysis of factors associated with advanced fibrosis.

Variable	Overall		Normal Weight		Overweight		Obese	
	OR 95% CI	*p*-Value	OR 95% CI	*p*-Value	OR 95% CI	*p*-Value	OR 95% CI	*p*-Value
Female gender	–	–	0.19(0.07-5.21)	0.32	–	–	–	–
BMI	1.05 (0.97–1.14)	0.20	–	–	–	–	1.1 (0.94–1.3)	0.09
Waist circ.	1.01 (0.97–1.04)	0.59	–	–	–	–	1 (0.98–1.03)	0.8
AST	1.02 (1–1.04)	0.001	1.03 (1–1.6)	0.02	1.03 (1.01–1.05)	0.003	1.04 (0.99–1.2)	0.01
HbA1c	1.1 (0.94–1.3)	0.21	–	–	–	–	1.01 (0.97–1.08)	0.17
CAP	1 (0.99–1.01)	0.41	1.01 (0.98–1.03)	0.40	–	–	–	–
Severe steatosis	2.5 (1.5–3.1)	0.09	–	–	–	–	5 (1.5–31.4)	<0.0001

CI = confidence interval; OR = odds ratio.

**Table 7:** Differences between liver stiffness measurements (LSM), APRI, and FIB-4 scores among patients with mild and significant fibrosis (F ≤ 2) and those with advanced fibrosis (≥F3).

	F ≤ 2 (*n* = 430)	≥F3 (*n* = 104)	*p*-Value
LSM (kPa)	5.82 ± 1.60	12.48 ± 7.9	<0.0001
APRI	0.29 ± 0.17	0.44 ± 0.3	<0.0001
FIB-4	1 ± 0.15	1.39 ± 1	<0.0001

## Supplementary Materials

The following are available online at https://www.mdpi.com/2077-0383/9/4/1032/s1, Figure S1: Vibration controlled transient elastography (VCTE) and controlled attenuation parameter (CAP) from the Fibroscan^®^ device
