# Supplementary material for: Screening for Liver Fibrosis and Steatosis in a Large Cohort of Patients with Type 2 Diabetes Using Vibration Controlled Transient Elastography and Controlled Attenuation Parameter in a Single-Center Real-Life Experience
Source: J Clin Med. 2020 Apr 6;9(4):1032. doi: 10.3390/jcm9041032 (PMC7230646; doi:10.3390/jcm9041032)
Supplement: Supplementary file 1 [file jcm-09-01032-s001.pdf]

Supplementary Materials

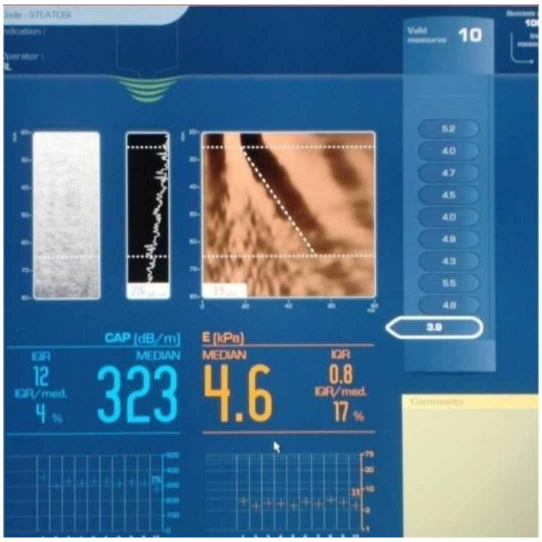

Figure S1. Vibration Controlled Transient Elastography (VCTE) and Controlled Attenuation Parameter (CAP) from the Fibroscan® device.
